# Metastatic Calcinosis Cutis in the Emergency Department: A Case Report

**DOI:** 10.21980/J87Q00

**Published:** 2025-07-31

**Authors:** Christian Hernandez-Zegada, Holly Conger, Brian Milman

**Affiliations:** * University of Texas Southwestern Medical Center, Department of Emergency Medicine, Dallas TX

## Abstract

Calcinosis cutis is a condition in which calcium-phosphate salt deposits are formed in cutaneous and subcutaneous tissue. A subtype, metastatic calcinosis cutis, can occur in patients with disorders that cause hypercalcemia or hyperphosphatemia such as end stage renal disease. We present a case of a 67-year-old man with end-stage renal disease (ESRD) on dialysis who presented to the emergency department with a draining left elbow wound. On exam, he had irregular, firm nodules palpable in the subcutaneous tissue of both large and small joints. The presence of calcinosis cutis on imaging and lack of other findings suggesting infection led to outpatient wound care treatment. Recognizing the appearance of calcinosis cutis on imaging and conditions that present with calcinosis cutis is important for the emergency physician.

**Topics:**

Calcinosis cutis, end-stage renal disease, ESRD, dialysis, subcutaneous calcifications.

**Figure:**
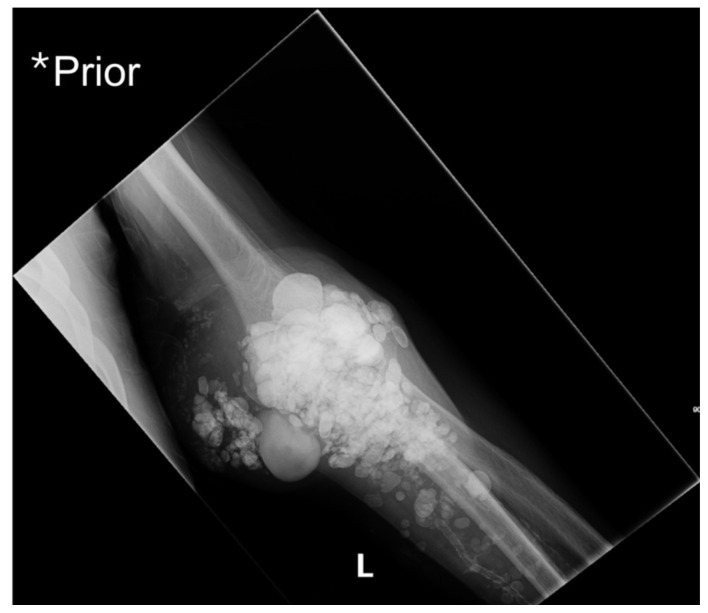


**Figure:**
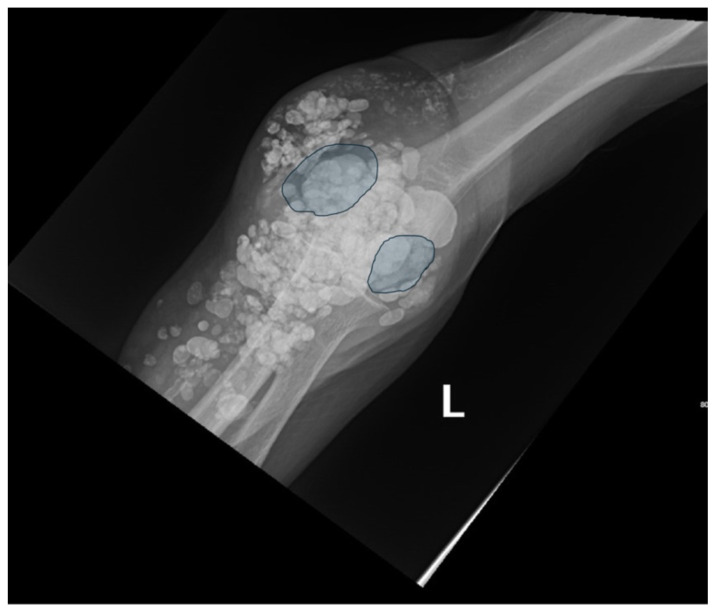


**Figure:**
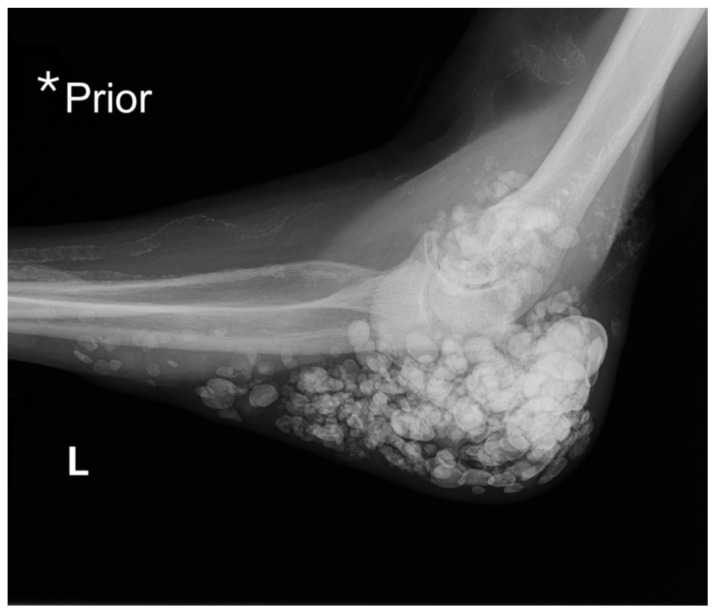


**Figure:**
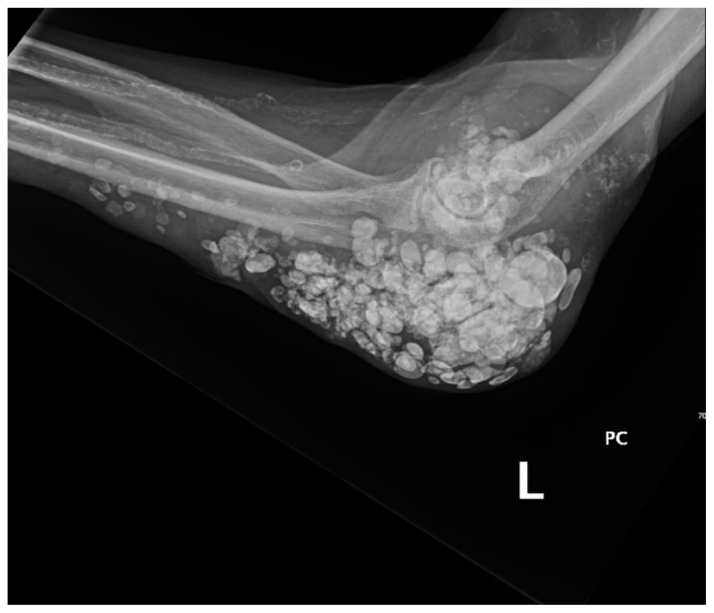


## Brief introduction

Calcinosis cutis is a condition in which insoluble calcium-phosphate salts are deposited in cutaneous and subcutaneous tissue. The five main subtypes include dystrophic, metastatic, idiopathic, iatrogenic, and calciphylaxis calcinosis cutis. Metastatic calcinosis cutis is characterized by abnormal calcium or phosphorus metabolism resulting in abnormal serum calcium or phosphorus levels.^1^ The prevalence of calcinosis cutis in an ESRD population is 0.5% – 1.2% and in dialysis patients, it can be as high as 7.0%.^2^,^3^ Many disorders are associated with metastatic calcinosis cutis, including sarcoidosis, Albright hereditary osteodystrophy, neoplasms, and hypervitaminosis D; however, it has the highest association with ESRD.^1^ Typical symptoms include pain, joint stiffness, nerve compression, and inflammation.^4^ Here, we present a case of metastatic calcinosis cutis in a dialysis patient and the associated radiographic findings.

## Presenting concerns and clinical findings

A 67-year-old male with a past medical history of ESRD on dialysis, benign prostatic hypertrophy, gout, hypothyroidism, and hypertension presented to the emergency department (ED) for routine dialysis. Due to insurance status, he presents to the ED for dialysis as needed rather than to a scheduled clinic. He is dialyzed once per week and completed dialysis one week prior to this visit. The patient denied chest pain, shortness of breath, or fevers. He had no difficulty with range of motion. He reported pain and drainage from his left elbow without preceding trauma or injury. He had been keeping the wound clean and covered due to the drainage. The patient reported that the pain was worse before it started spontaneously draining a few days prior. He noted that his elbow and other large joints had been swollen for a long time, but he had never had one of them drain before.

## Significant findings

On physical exam, the patient was found to have diffuse, asymmetrical, firm, round nodules underneath the skin at most joints including large and small joints of the upper and lower extremities. The nodules were most pronounced in his hands and elbows. Over the lateral left elbow there were two punctate wounds that were open and draining a thick, chalky, white material that contained sediment. There was no surrounding erythema or warmth, and the elbow itself was not tender to the touch. All joints had full range of motion without pain.

The patient’s temperature was 36.8 degrees Celsius, heart rate was 64 BPM, and blood pressure was 138/92 mmHG. His oxygen saturation was normal on room air. His labs were notable for an elevated potassium (5.5 mmol/L), normal calcium (8.8 mg/dL), and elevated phosphorus (8.6 mg/dL). Additionally, he had elevated inflammatory markers with a C-reactive protein (CRP) of 5.9 mg/dL and erythrocyte sedimentation rate (ESR) of 22 mm/hr. Electrocardiograph (ECG) showed no arrhythmia and no electrocardiographic signs of hyperkalemia.

X-ray imaging was obtained of the left elbow and showed soft tissue calcium deposits. Radiology stated, “massive periarticular calcinosis of renal failure obscures fine osseous detail. Several of the largest calcifications have decompressed since the prior exam and may contribute to the drainage observed clinically. Superimposed infection is not excluded.” X-rays with an asterisk are the comparison images from two months previous to the visit. Areas of decompression are highlighted in blue demonstrating that some of the larger calcified nodules are no longer present.

## Patient Course

In the setting of draining wounds and elevated ESR and CRP, soft tissue infection and septic arthritis were on the differential. However, ESR and CRP are non-specific, especially in a patient with ESRD.^5^ Additionally, the patient had no systemic signs of infection, including no erythema or warmth overlying or surrounding the area of drainage, no fever, and no pain with active or passive range of motion of the joint. As a result, infection was excluded clinically as a cause of the patient’s symptoms.

Given the decompression of deposits when compared to previous imaging, we felt that the patient’s drainage consisted of calcium deposits that had eroded through the skin and decompressed, rather than an infectious etiology. The patient was admitted to the nephrology service for routine dialysis and discharged with follow-up in an ED follow-up clinic so the area of drainage could be closely monitored. When the patient subsequently followed up in clinic, he continued to have drainage but had no signs of infection or significant change in exam. The patient has been seen in the ED multiple times since and has never developed joint or soft tissue infection.

## Discussion

We identified calcinosis cutis in a patient with ESRD and hyperphosphatemia. Dialysis patients are a high morbidity population that are often encountered in the ED. Although rare, metastatic calcinosis cutis can be seen in this population, and an understanding of the physical exam and lab findings in these patients is important. If subcutaneous nodules and X-ray findings of calcinosis cutis are identified in patients with normal calcium and phosphorus levels, other subtypes of calcinosis cutis, specifically dystrophic calcinosis cutis, must be considered and may indicate underlying systemic disease.^6^ Dystrophic calcinosis cutis can occur in scleroderma, dermatomyositis, systemic lupus erythematosus, and porphyria. If present in a patient with no previous diagnosis, additional outpatient rheumatologic workup is required.

Our patient had drainage from his left elbow that we attributed to draining calcium salts rather than infection. There are few case reports of calcinosis cutis mimicking skin infection.^7^,^8^ There are no guidelines or randomized trials to guide therapy in this complex patient population. In case reports, antibiotic use was variable. In our case, the radiographic improvement in calcifications contributed to our decision not to treat with antibiotics. Normally, X-ray imaging of metastatic calcinosis cutis is characterized by periarticular, lobulated calcifications within the soft tissue, often affecting extensor surfaces.^9^ Although sensitivity and specificity of X-ray is unknown, X-ray is effective at identifying the extent of tissue involvement, the pattern of distribution, which is helpful in determining the subtype, and at excluding other differential diagnoses. Therefore, if calcinosis cutis is suspected and has not been previously diagnosed, imaging is an essential part of the diagnostic workup.^1^

Management of metastatic calcinosis cutis primarily involves management of the cause of the metabolic derangement. In this case, where the underlying condition was ESRD, treatment includes regular dialysis and close monitoring of labs. Because of our patient’s complicated social situation, he was receiving weekly dialysis rather than dialyzing three times weekly which contributed to his hyperphosphatemia.

A case series that included 42 patients with calcinosis cutis demonstrated improvement of superficial lesions in 78% of patients treated with sodium thiosulfate, but most of the cases were dystrophic calcinosis cutis, and patients were not followed beyond six months.^10^ Other therapies, such as diltiazem, colchicine, and minocycline have mixed benefits in dystrophic and autoimmune causes of calcinosis cutis and have not been evaluated in metastatic calcinosis cutis. Finally, surgery has been used as an adjunct treatment for large, discrete, and symptomatic regions of deposits to provide relief and functional improvement, but there is insufficient evidence that lesions should be incised and drained in the ED.^11^

An understanding of the disease process, labs, and imaging findings of metastatic calcinosis cutis is relevant to the emergency physician because ESRD patients are often encountered in the ED. Calcinosis cutis may also be found incidentally on imaging; thus, awareness of imaging findings is important. If the etiology cannot be immediately identified in the ED, patients should be referred for additional outpatient workup.

## Supplementary Information












