# Supplementary figures and images for: Metastatic Calcinosis Cutis in the Emergency Department: A Case Report
Source: J Educ Teach Emerg Med. 2025 Jul 31;10(3):V1–4. doi: 10.21980/J87Q00 (PMC12321003; doi:10.21980/J87Q00)

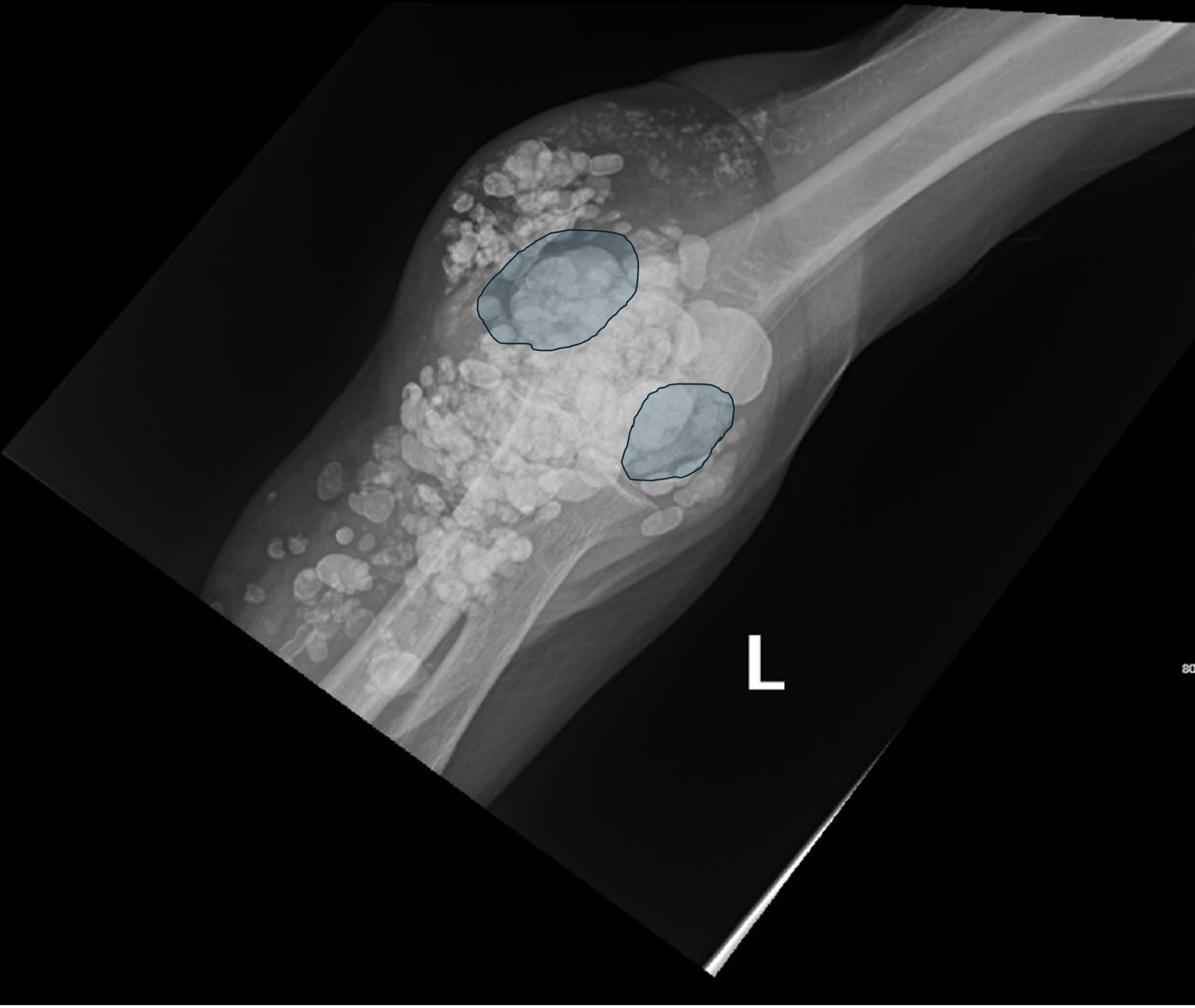

Supplement: Supplementary file 1 [file 10-3-V1-Supp1.jpg]

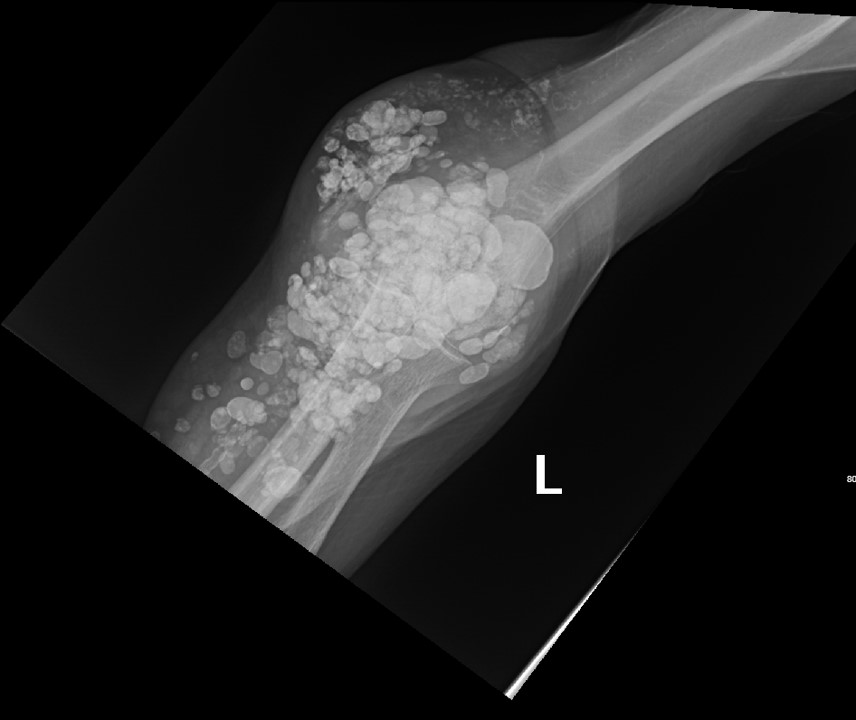

Supplement: Supplementary file 2 [file 10-3-V1-Supp2.jpg]

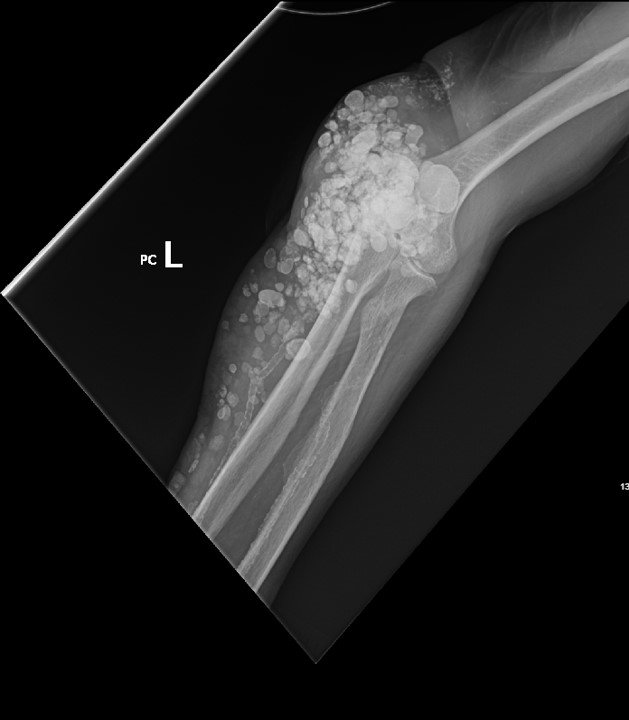

Supplement: Supplementary file 3 [file 10-3-V1-Supp3.jpg]

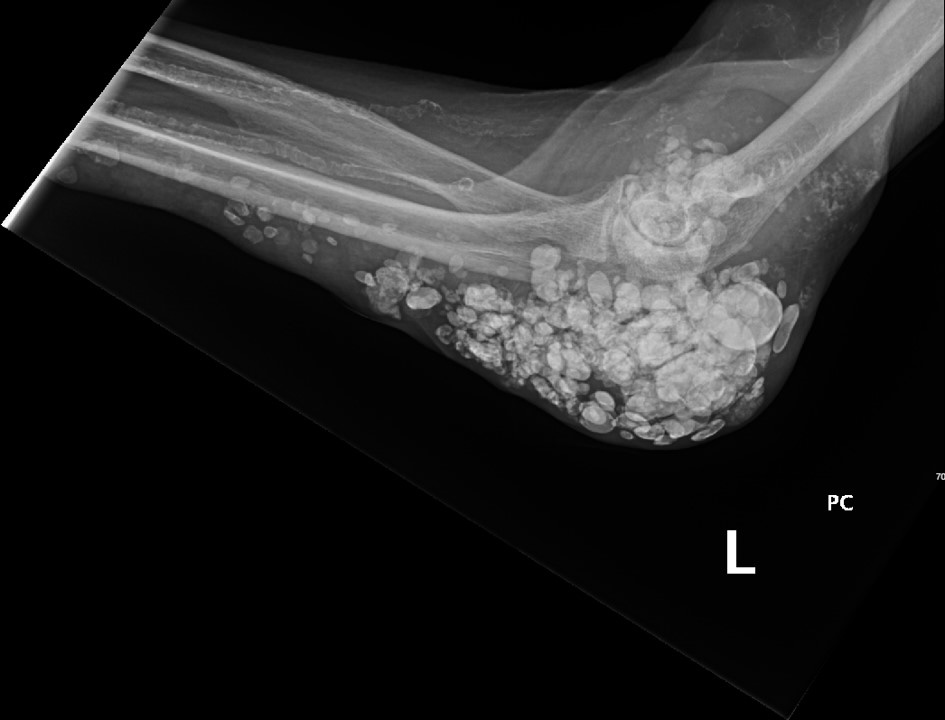

Supplement: Supplementary file 4 [file 10-3-V1-Supp4.jpg]

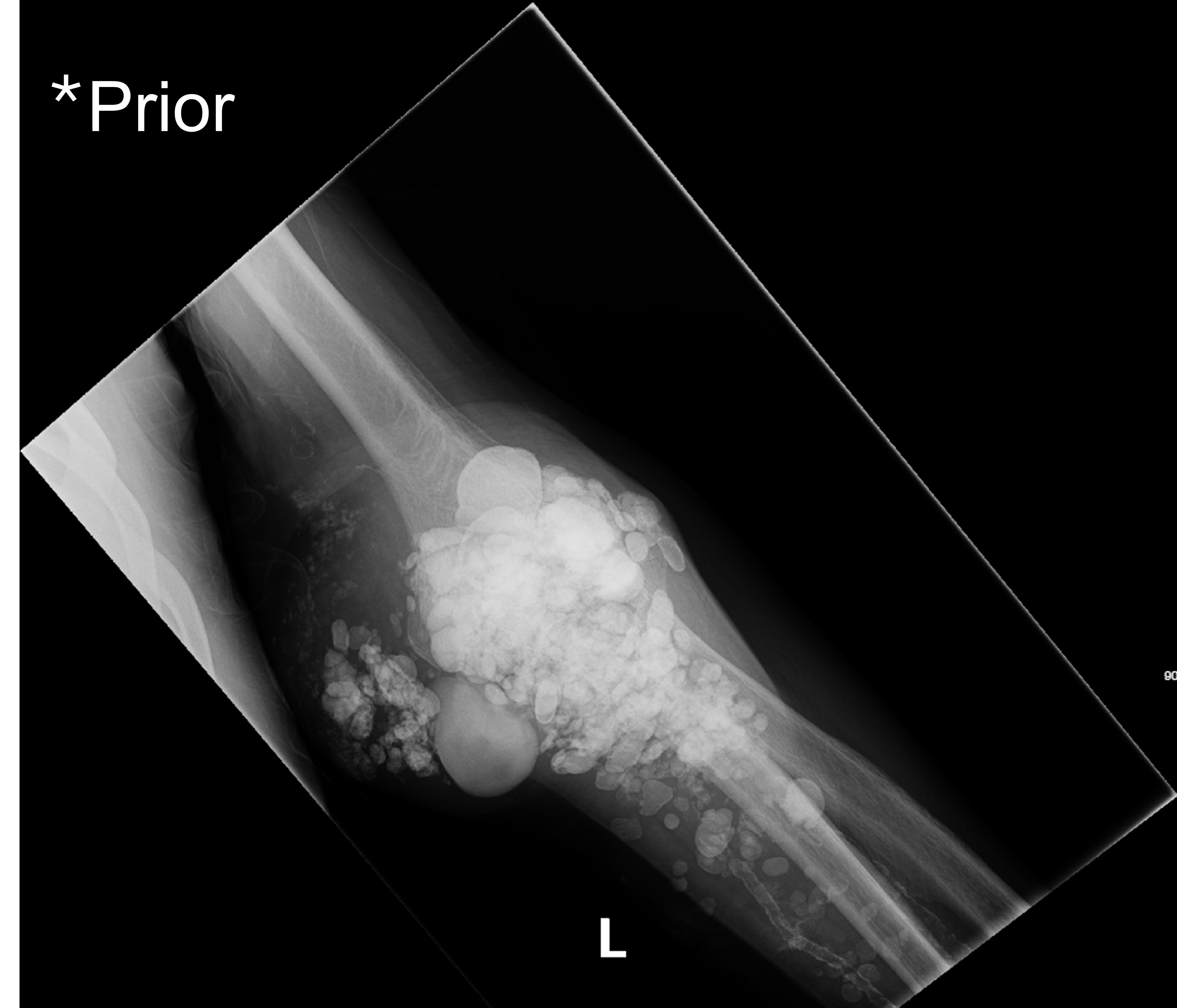

Supplement: Supplementary file 5 [file 10-3-V1-Supp5.jpg]

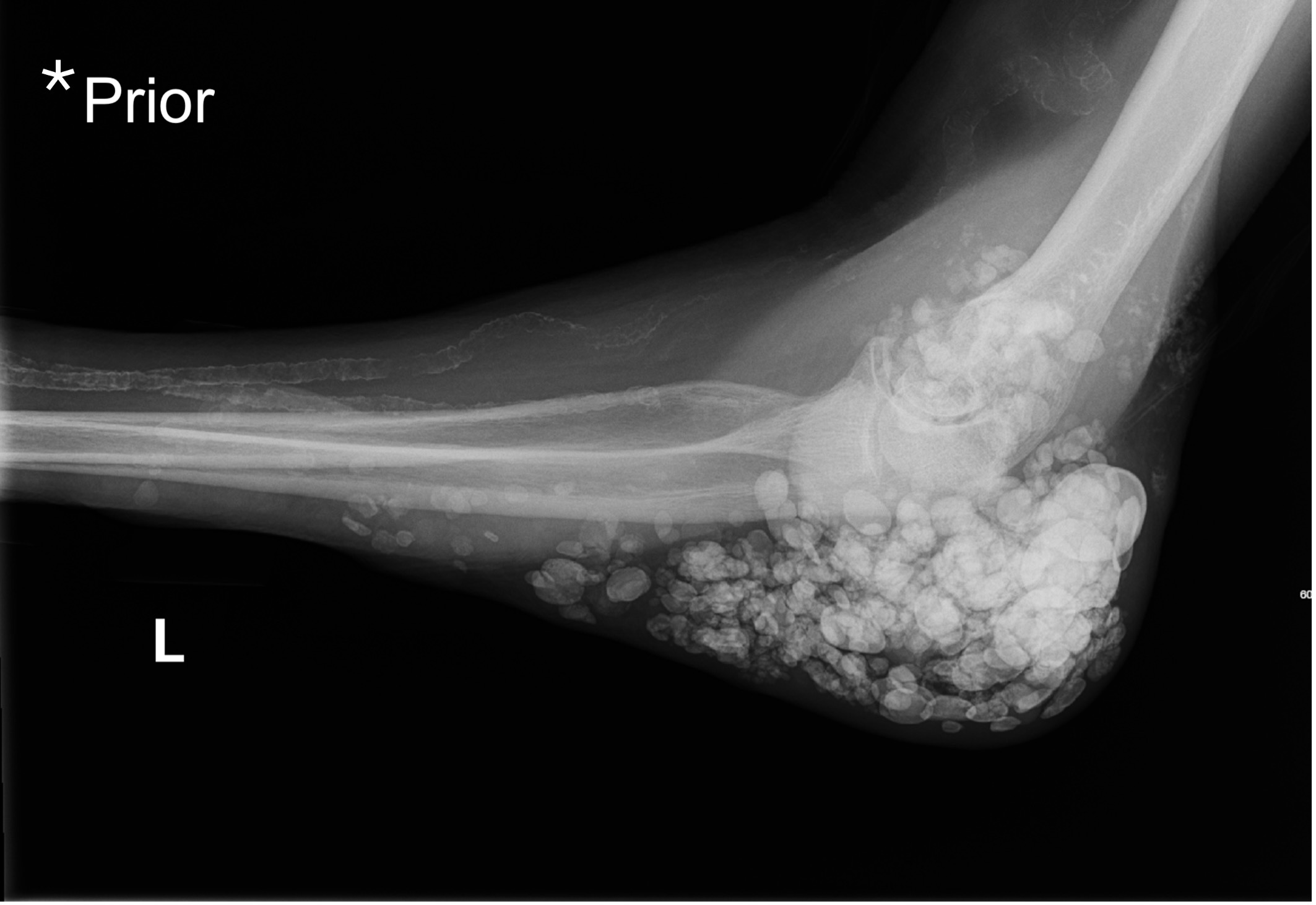

Supplement: Supplementary file 6 [file 10-3-V1-Supp6.jpg]
